# Prevalence of ADHD in FND

**DOI:** 10.1192/bjo.2026.11135

**Published:** 2026-06-30

**Authors:** Saranjan Manivannan, Leo Nihoyannopoulos, Bruce Tamilson

**Affiliations:** 1 UCL, London, United Kingdom; 2 South West London and St George’s NHS Trust, London, United Kingdom; 3 City St George’s University, London, United Kingdom

## Abstract

**Aims:**

Growing research indicates that attention-deficit/hyperactivity disorder (ADHD) may occur at higher rates in individuals with functional neurological disorder (FND). However, reported prevalence differs substantially across studies and FND subtypes. This systematic review and meta-analysis sought to estimate the frequency of ADHD comorbidity within FND populations.

**Methods:**

A systematic literature search of PubMed, Embase, Web of Science, PsycINFO, and Google Scholar was conducted through October 2025. Seventy-five studies fulfilled inclusion criteria. Information was extracted regarding study design, sample characteristics, diagnostic methods, and reported ADHD prevalence. Pooled prevalence estimates were calculated using random-effects meta-analytic models.

**Results:**

Across 63 studies involving 115,331 participants, the pooled prevalence of ADHD among individuals with FND was 15% (95% CI: 12–19; I²=92%). Subgroup analyses indicated ADHD rates of 14% (95% CI: 10–18) in functional seizures (22 studies, n=11,117), 6% (95% CI: 1–16) in functional movement disorder (4 studies, n=166), and 27% (95% CI: 19–35) in functional tic-like behaviours (21 studies, n=1057). In studies limited to paediatric populations (33 studies, n=2141), the pooled prevalence was 16% (95% CI: 11–22).

**Conclusion:**

Approximately one in six individuals with FND also meet criteria for ADHD, with particularly elevated rates observed in functional tic-like presentations and in children and adolescents. Considerable heterogeneity was present across studies. These findings underscore the importance of evaluating for ADHD during FND assessment, especially when difficulties with attention regulation or impulse control are prominent.

